# Survey of the triple-mentoring program for students at a religious medical school

**DOI:** 10.1186/s12909-021-02593-z

**Published:** 2021-03-16

**Authors:** Ting-Chun Tseng, Tsung-Ying Chen, Shao-Yin Chu, Hung-Che Wang, Ching-Yuan Chang

**Affiliations:** 1 School of Medicine, Tzu Chi University, Hualien, Taiwan; 2 Department of Medical Education, Buddhist Tzu Chi General Hospital, Hualien, Taiwan; 3 Department of Anesthesiology, Buddhist Tzu Chi General Hospital, Hualien, Taiwan; 4 Department of Pediatrics, Buddhist Tzu Chi General Hospital, Hualien, Taiwan; 5 Department of Education and Human Potentials Development, National Dong Hwa University, Hualien, Taiwan; 6 Institute of Education, Tzu Chi University, Hualien, Taiwan

**Keywords:** Mentoring program, Guidance, Counseling, Humanity

## Abstract

**Background:**

Tzu Chi University in Taiwan offers a unique mentoring program. This program differs from others as it comprises triple mentorship, namely, faculty mentors, Tzu Cheng/Yi De (TC/YD; senior volunteers), and school counselors. This study aimed to survey the role functions of the mentors from the perspective of medical students.

**Methods:**

The Role Functions of the Mentoring Program Scale (RFMPS) was developed on the basis of literature reviews and focus groups and it underwent exploratory factor analysis for internal consistency and reliability. RFMPS comprises four role functions, namely, mental, educational, career, and humanistic/moral guidance counseling. The survey was distributed to 171 medical students via an online network with two-month intervals and was analyzed using multivariate analysis of variance.

**Results:**

The overall response rate was 64% (116/171). The mean scores of the four role functions in descending order belonged to faculty mentors, TC/YD, and school counselors. For humanistic/moral guidance, students had an equal preference for the faculty mentors and TC/YD over school counselors. As for educational, career, and mental guidance counseling, students preferred faculty mentors over TC/YD and school counselors. Faculty mentors provided students with the required guidance counseling for all the four role functions, especially educational guidance; TC/YD in particular offered prominent humanistic/moral guidance and career counseling; school counselors were less preferred but guided students in need.

**Conclusions:**

Medical students value different role functions provided by faculty mentors, TC/YD, and school counselors. A diversified focus could be provided by the faculty mentors, particularly in educational, career, mental, and humanistic/moral counseling; TC/YD specialized in humanistic/moral guidance; and the school counselors carried out their role function only when needed. Humanistic/moral guidance is equally preferred to other types of guidance, which can be equally valuable in future mentoring programs.

**Supplementary Information:**

The online version contains supplementary material available at 10.1186/s12909-021-02593-z.

## Background

A mentor is a senior who responds to mentees’ questions, gives optimal advice, shares information, listens actively, and stimulates students’ reflection [1–4]. There are many types of mentoring programs, such as dual-mentoring, peer-mentoring, group-based mentoring, and personalized mentoring [5–7], and most of them are longitudinal throughout the medical curriculum. Mentoring programs not only provide an immediate support network but also address several important themes, such as professionalism, empathy, patient-centered care, cultural sensitivity, collaboration, ethical decision-making, altruism, honor and integrity, respect, and accountability. An effective mentoring program is one of the most important features of higher education because it not only helps students understand themselves and their world but also facilitates personal development [7–14].

In this regard, Tzu Chi University (TCU) in Taiwan offers a unique, triple-mentoring program, which includes faculty mentors, Tzu Cheng/Yi De (TC/YD, who are senior volunteers), and school counselors. There were 15 licensed and appointed school counselors at TCU (counselor–student ratio, 1:225), and their major services comprised individual consultation, group consultation, and psychological testing for students in need [15, 16]. Students could make an appointment for counseling when needed. Faculty mentors are teachers who are required to mentor students first, followed by faculty development programs as well as participating in regular mentor society meetings. All mentors are supported by the administrative body.

**Fig. 1:**
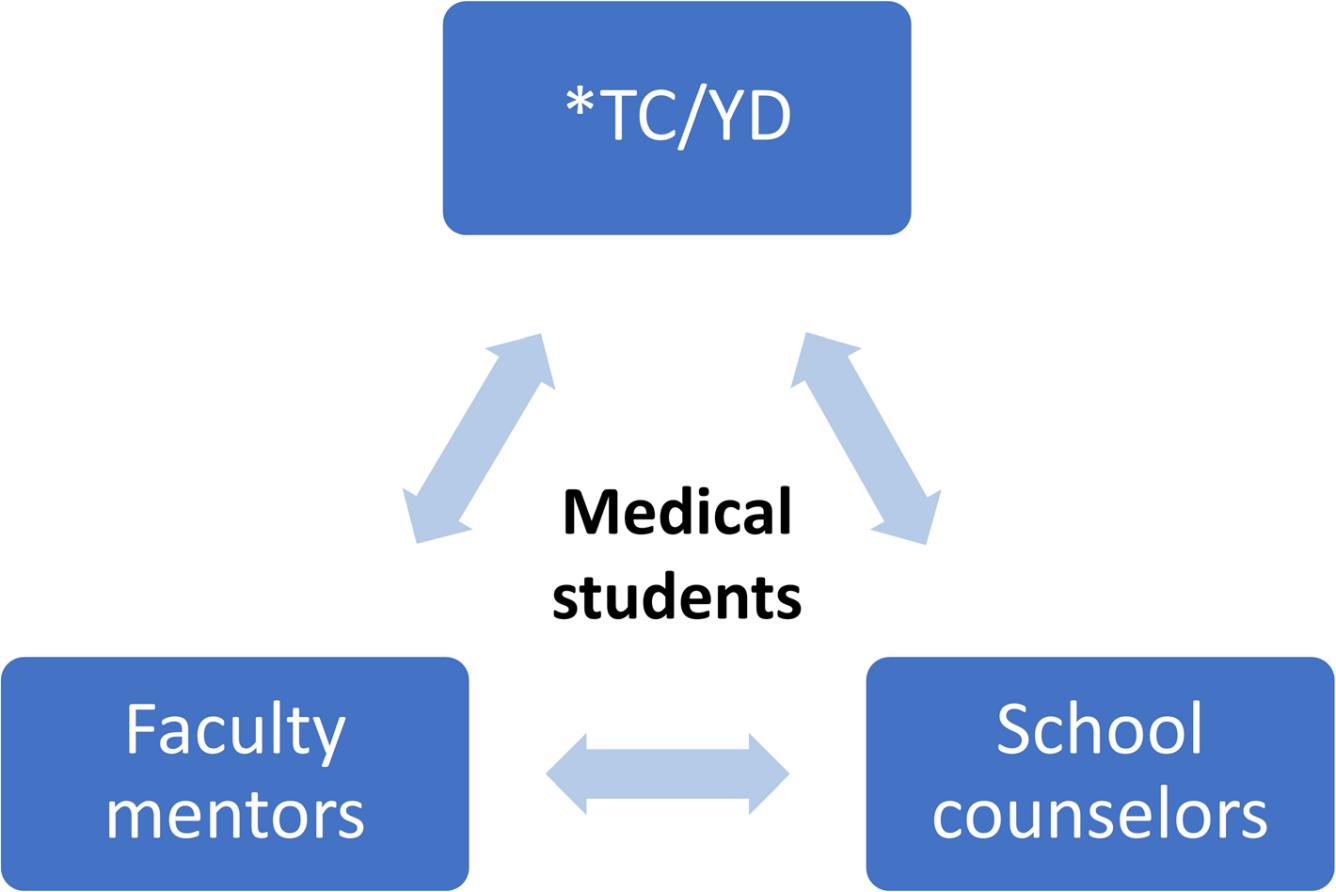
The triple mentorship at Tzu Chi University. *TC/YD (Tzu Cheng/Yi De): senior volunteers with kindness and enthusiasm to medical education, and domain experts in various fields

TC/YD is a exclusive program that differs from conventional mentoring systems. TC/YD participants’ contribution is as per the school’s mission statement: to prepare those who embrace “humanistic literacy” and are willing to assist those in need. TC/YD are senior volunteers who are subject-matter experts in various fields, such as professors, doctors, lawyers, civil servants, and businesspersons; they are selected and appointed to provide students with humanistic and moral guidance by the institution [17]. Three to four TC/YD, 1 faculty mentor, and 10–13 students meet monthly (approximately four times per semester) for various humanistic activities. For instance, they participate in the “tea ceremony,” which is an annual activity wherein medical students serve tea to their teachers, symbolizing the students’ respect and gratitude. Additionally, TC/YD share their life stories, valuable philosophies, major life events, and ideals of volunteerism and altruistic behavior. Because most TC/YD are vegetarians and environmentalists, regular topics during the monthly gathering often involve recycling, environmental protection, and low-carbon footprint diet or vegetarianism. Furthermore, at these monthly gatherings, participants consume vegetarian food and avoid using disposable tableware, and most importantly, the objective behind is to maintain this as a convention for the ceremony and internalize it within each participant as a virtuous habit in the long run. In summary, TC/YD promotes positive characteristics and humanistic behaviors in interpersonal, social, and environmental relationships [18–21]. The triple mentorship is shown in Fig. 1.

Although TC/YD have been part of TCU’s mentoring program since 1994, the program was not well known. This study surveys student opinions and introduces new dynamics to the mentoring program. This study aimed to explore ideas and opinions of medical students in the triple-mentoring system, and assess role function items through literature review as well as focus group method analysis aided with the development of assessment tools.

## Methods

### Development of survey scale

The draft Role Functions of the Mentoring Program Scale (RFMPS) was developed on the basis of literature reviews and a series of heterogeneous focus groups to reach a consensus. The focus groups comprised medical students and expert faculty mentors, TC/YD, and school counselors [22, 23]. An additional documentation file shows the focus group discussion guide in English (Additional file 1). The draft items were initially categorized into five role functions, namely, life, mental, educational, career, and humanistic/moral guidance counseling. The items were then validated by six qualitative research experts for content and clarity of wording. The Kaiser-Meyer-Olkin Test and Bartlett’s Test of Sphericity were conducted to measure sample adequacy and statistical requirements for exploratory factor analysis. The primary component method with varimax orthogonal rotation was tested, including tests for items with eigenvalues (> 1) and excluding items with factor loading (< 0.4) [24, 25]. Finally, the exploratory factor analysis extracted four role functions by excluding life guidance from the five role functions. The RFMPS had good internal consistency and reliability, with an acceptable total explanation of variance ranging from 76.29 to 85.62% and an excellent Cronbach’s alpha ranging from .95 to .97 [25]. The RFMPS in Chinese is a 16-item tool that measures the role functions of the three types of mentors on a 5-point Likert scale, ranging from 1 (strongly disagree) to 5 (strongly agree). Additional file 2 presents the English Version of the RFMPS.

### Setting and respondents

The survey was distributed via social networks, and 171 medical students were invited to take the survey 2 months into the fall semester of 2019. Online surveys were distributed, and RFMPS was delivered to medical students (from first to sixth year) in two-month intervals.

### Data analysis

All data were analyzed using SPSS Statistics for Windows, version 18.0 (Armonk, NY). Descriptive analysis of the triple-mentorship role functions was performed. Multivariate analysis of variance (MANOVA) was conducted to analyze each role function of three types of mentors and the four role functions among them.

## Results

### Demographics and descriptive statistics

The response rate was 64% (116/171). The respondent data included gender, undergraduate year, nationality, religion, frequency of contacting the three types of mentors are presented in Table 1. The undergraduate year of respondents ranged from first to sixth year, with 54.3% men and 45.7% women. A diverse pool of TCU medical students, both international and domestic ones from all parts of Taiwan were surveyed, and the five main religions of respondents were Buddhism, Taoism, Chinese folklore religion, Christianity, and Shamanism. The type of mentor contacted by students in descending order of frequency of contact was TC/YD, faculty mentors, and school counselors; and the percentages of students who never contacted faculty mentors or TC/YD was low (16.4 and 4.3%, respectively), except for noncontact with the school counselor (59.5%). In general, the mean scores of four role functions in descending order were faculty mentors, TC/YD, and school counselors (Table 2). Regarding humanistic/moral guidance, in particular, faculty mentors had the highest mean scores (M = 3.82, SD = .70), followed by TC/YD (M = 3.79, SD = .95) and school counselors (M = 2.86, SD = 1.14).

**Table 1:** Demographics of respondents

Variables	Total (*N* = 116)	
	N	Percentage
Gender		
Men	63	54.3
Women	53	45.7
Undergraduate year		
First	9	7.7
Second	27	23.3
Third	32	27.6
Fourth	34	29.3
Fifth to sixth	14	12.1
Nationality		
Native	110	94.8
Non-native	6	5.2
Religion		
Irreligion	73	62.9
Buddhism	17	14.7
Taoism	10	8.6
Chinese folk religion	8	6.9
Christianity	7	6.0
Shamanism	1	0.9
Frequency of communication with faculty mentors per semester		
Thrice or more	47	40.5
Once or twice	50	43.1
Never	19	16.4
Frequency of communication with Tzu Cheng/Yi De per semester		
Thrice or more	83	71.6
Once or twice	28	24.1
Never	5	4.3
Frequency of communication with school counselors this semester		
Thrice or more	11	9.5
Once or twice	36	31.0
Never	69	59.5

**Table 2:** Descriptive statistics of the RFMPS with possible score of 1–5 (*N* = 116)

Role function	Faculty mentors		TC/YD		School counselors	
	M	SD	M	SD	M	SD
Mental counseling	3.65	.80	3.26	.99	2.86	1.18
Educational guidance	4.04	.71	2.93	.98	2.51	1.06
Career counseling	3.87	.79	3.44	.97	2.63	1.12
Humanistic/moral guidance	3.82	.70	3.79	.95	2.86	1.14

*Abbreviations: M* mean, *SD* standard deviation

### Posteriori test for each role function between the three types of mentors

MANOVA analysis, as indicated in Table 3, showed statistically significant multivariate differences (Wilk’s Λ = 37.23, *P* < .001). The univariate F test and the posteriori test suggested that there were significant differences between the three types of mentors: both faculty mentors and TC/YD had higher scores than the school counselors for humanistic/moral guidance (F _(2,345)_ = 38.88, *P* < .0125); faculty mentors had higher scores than TC/YD, and TC/YD had higher scores than school counselors for mental counseling (F _(2,345)_ = 18.17, *P* < .0125), educational guidance (F _(2,345)_ = 83.72, *P* < .0125), and career counseling (F _(2,345)_ = 48.92, *P* < .0125).

**Table 3:** MANOVA summary table for each role function between the three types of mentors

Variables	*df*	SSCP				Multivariate	Univariate *F*-ratio			
						*Wilk’s Λ*	Mental counseling	Educational guidance	Career counseling	Humanistic/ moral guidance
Between subjects	2	36.18	69.32	57.06	44.60	37.23***	18.17*	83.72*	48.92*	38.88*
		69.32	143.59	104.46	72.90					
		57.06	104.46	92.16	75.98					
		44.60	72.90	75.98	69.59					
Within subjects	345	343.54	243.28	285.38	252.24					
		243.28	295.85	250.30	219.28					
		258.38	250.30	327.99	242.02					
		252.24	219.28	242.02	308.75					
Posteriori Test							*FM* > *TC/YD* > *SC*	*FM* > *TC/YD* > *SC*	*FM* > *TC/YD* > *SC*	*FM*, *TC/YD* > *SC*

*Abbreviations: FM* faculty mentors, *TC/YD* Tzu Cheng/Yi De, *SC* School counselors, *df* degree of freedom

*p value < .0125

***p value < .001

### Posteriori test for the four role functions among each mentor

The MANOVA analysis (Table 4) showed statistically significant multivariate differences (Wilk’s Λ = 11.24, *P* < .001).

**Table 4:** MANOVA summary table for the four role functions among each mentor

Variables	*df*	SSCP			Multivariate	Univariate *F*-ratio		
					*Wilk’s Λ*	Faculty mentors	TC/YD	School counselors
Between subjects	3	8.94	−8.29	−8.33	11.24***	5.29*	15.92*	2.72
		−8.29	45.06	14.94				
		−8.33	14.94	10.29				
Within subjects	460	259.16	165.09	95.79				
		165.09	434.16	169.49				
		95.79	169.49	579.80				
Posteriori Test						*EG* > *MC*	*CC* > *EG*; *HG* > *MC*, *EG*	

*Abbreviations: MC* mental counseling, *EG* educational guidance, *CC* career counseling, *HG* humanistic/moral guidance, *df* degree of freedom

*p value < .0167

***p value < .001

The univariate F test and the posteriori test suggested that there were statistically significant differences among faculty mentors: educational guidance had higher scores than mental counseling (F _(3,460)_ = 5.29, *P* < .0167); and between TC/YD, career counseling had higher scores than educational guidance, and humanistic/moral guidance had higher scores than mental counseling and educational guidance (F _(3,460)_ = 15.92, *P* < .0167). However, there were no significant differences among school counselors (F _(3,460)_ = 2.72, *P* > .0167).

## Discussion

This study was the foremost quantitative study that surveyed the triple-mentoring program at a religious medical school, analyzing medical students’ perspectives regarding the role functions of faculty mentors, TC/YD, and school counselors. Tables 3 and 4 indicated that the three types of mentors had distinguished role functions. Overall, faculty mentors offered medical students guidance counseling that was most preferred in all four role functions, particularly in educational guidance; and TC/YD provided humanistic/moral guidance and career counseling that was preferred and prominent. Conversely, school counselors were less preferred.

Table 3 shows how medical students perceived supportive guidance from faculty mentors. This affirms that the frequency and nature of guidance were positively correlated with mentors’ supportiveness and program satisfaction in previous studies [26, 27]. This finding is also in line with related studies on how faculty mentors can have a positive impact on students through various approaches [1–4]. The fact that medical students gave low scores to school counselors should be interpreted cautiously (Table 3). In this regard, such scores could have been based on the low counselor–student ratio and less contact or demand with school counselors (Table 1). The most significant result of this study was that TC/YD provided students with powerful humanistic/moral guidance and career counseling, which are novel and important. Most students were willing to contact TC/YD at the monthly gathering at least three times a semester for humanistic/moral guidance and career counseling (Table 1). This was probably because the TC/YD were senior volunteers with regard for kindness and enthusiasm in education as well as had subject-matter experts in various fields. They were willing to maintain a close connection with the students, share their life stories, experiences, and philosophy of life, which affirms TCU’s mission statement, that is, to prepare those who embrace “humanistic literacy” and to assist those in need [18, 21]. As aforementioned, TC/YD gained themselves a position in the mentoring program and provided comprehensive guidance along with the faculty mentors and school counselors.

Although few tools have been conceptualized humanity and morality as part of the mentoring programs [13, 28], the mean scores of the four role functions shown in Tables 3 and 4 indicate that medical students equally preferred humanistic/moral guidance and other types of guidance. Humanistic/moral guidance can be interpreted as guidance that promotes or enlightens one’s positive characteristics and behaviors in interpersonal, social, and environmental relationships, which differs from the conventional aim of mentoring programs that focus on aspects such as career, psychosocial, personal/emotional function, pedagogical knowledge, professional development, interpersonal problems, and role modeling. Therefore, one may question whether the content of mentoring is different or whether the perception of students toward mentoring varies in the post-millennium era. From the system level, findings suggested that the development of a mentoring system should not only focus on the conventional content of education, career, and emotion but should also equally value humanity, morality, and relationships.

There are several limitations worth noting. This study only presents medical student’s perspectives of the triple-mentoring program, which cannot evaluate other functions of mentoring systems, such as the effect of strategies in enhancing mentorship, outcome of medical student’s career development, and influence of subject-matter experts. Because TC/YD were volunteers and unpaid social elites, it is difficult to perceive that such a program could be applicable in other schools. To our knowledge, there is no other institution that provides triple mentorship, particularly senior volunteers and the implementation of humanistic/moral guidance. Owing to the exclusive quality of the triple-mentoring program, all outcomes were based on medical students at TCU. Despite these limitations, RFMPS can still be selectively utilized for faculty mentors and school counselors. Thus, future research could investigate additional insights into mentoring programs and humanistic/moral guidance using in-depth interviews or focus groups, explore the utilization of the RFMPS on a wider scale, and incorporate the humanistic/moral aspects of mentoring programs.

## Conclusions

This study surveyed the role functions of triple mentorship from the perspective of medical students. The three types of mentors had distinguished role functions. Faculty mentors contributed to educational, career, mental, and humanistic/moral guidance counseling; TC/YD specialized in humanistic/moral guidance; and school counselors carry out their role functions only when they were needed. Humanistic/moral guidance can be a rising concern for mentoring programs and is equally important to academic-related guidance such as educational, career, and mental guidance counseling.

## Supplementary Information

**Additional file 1.** The focus group discussion guide.

**Additional file 2.** The English version of the Role Functions of the Mentoring Program Scale.

## Data Availability

The survey tool used for the study is provided as supplementary information. All data are stored according to the ethical standards and are available upon reasonable request to the authors.

## Abbreviations

TCU: Tzu Chi University
TC/YD: Tzu Cheng/Yi De
RFMPS: Role Functions of the Mentoring Program Scale
MANOVA: Multivariate analysis of variance
