# Supplementary material for: Survey of the triple-mentoring program for students at a religious medical school
Source: BMC Med Educ. 2021 Mar 16;21:159. doi: 10.1186/s12909-021-02593-z (PMC7970779; doi:10.1186/s12909-021-02593-z)
Supplement: Supplementary file 1 — Additional file 1. The focus group discussion guide. [file 12909_2021_2593_MOESM1_ESM.docx]

Additional File 1

Focus Group Discussion Guide

| 10 min | *Introduction and welcome*  Introduction of names and roles of attendees:  The focus group discussion aims to gain insight about triple mentorship from the perspectives of medical students, faculty members, and school counselors, as well as Tzu Cheng/Yi De. It aims to develop items for the Role Functions of the Mentoring Program Scale.   - - Tell me about your first impression of triple mentorship in Tzu Chi University. |
| --- | --- |
| 60 min | *Discussion of main points*   1. Please share your thoughts on faculty mentors, Tzu Cheng/Yi De, or school counselors. 2. What are the advantages and disadvantages of triple mentorship? Please provide reasons for your answers. 3. Please describe the common or unique guidance counseling role functions of triple mentorship. 4. What are the important features or key elements of being a mentor?    - Personality    - Knowledge or proficiency |
| 15 min | *Discussion about experience*  Based on experience, please identify the strengths and challenges encountered during interactions with students?   - - What are the key factors? Why are these important?   - What have you done to overcome challenges?   - How can you build a professional relationship with mentees? Please briefly explain your answer.   - How do you feel about being a mentor to medical students? |
| 5 min | *Closure*   1. Is there anything else you would like to say? |
