# Supplementary material for: Survey of the triple-mentoring program for students at a religious medical school
Source: BMC Med Educ. 2021 Mar 16;21:159. doi: 10.1186/s12909-021-02593-z (PMC7970779; doi:10.1186/s12909-021-02593-z)
Supplement: Supplementary file 2 — Additional file 2. The English version of the Role Functions of the Mentoring Program Scale. [file 12909_2021_2593_MOESM2_ESM.docx]

Additional file 2. The English Version of the Role Functions of the Mentoring Program Scale

Possible scores: 1 (strongly disagree), 2 (disagree), 3 (neutral), 4 (agree), and 5 (strongly agree)

**Mental counseling**

I feel that my mentor is aware of my feelings.

My mentor guides me to manage my mental stress.

My mentor listens to my feelings when I am down.

I feel that my mentor empathizes with my mental struggles.

I share my feelings with my mentor when I am upset.

**Educational guidance**

My mentor gives me advice related to academics.

My mentor guides me on academic research.

My mentor knows my learning status.

I talk about courses and learning issues with my mentor.

**Career counseling**

My mentor is attentive to my career orientation.

I feel that my mentor guides my career development.

I discuss my future goals with my mentor.

**Humanistic/moral guidance**

My mentor influences me to be environmentally friendly.

My mentor motivates me to give without any expectations.

My mentor inspires me to have good manners and etiquette.

I learn positive character traits from the words and deeds of my mentor.
